# Characterization of the Cellular Output of a Point-of-Care Device and the Implications for Addressing Critical Limb Ischemia

**DOI:** 10.1089/biores.2015.0006

**Published:** 2015-11-01

**Authors:** Jennifer E. Woodell-May, Matthew L. Tan, William J. King, Matthew J. Swift, Zachary R. Welch, Michael P. Murphy, James M. McKale

**Affiliations:** ^1^ Biomet Biologics, LLC., A Subsidiary of Biomet, Inc., Warsaw, Indiana.; ^2^ Department of Surgery, Indiana University School of Medicine, Indianapolis, Indiana.

**Keywords:** autologous, CLI, EPC, HSC, MSC, peripheral arterial disease, point-of-care, stem cells

## Abstract

Critical limb ischemia (CLI) is a terminal disease with high morbidity and healthcare costs due to limb loss. There are no effective medical therapies for patients with CLI to prevent amputation. Cell-based therapies are currently being investigated to address this unmet clinical need and have shown promising preliminary results. The purpose of this study was to characterize the output of a point-of-care cell separator (MarrowStim P.A.D. Kit), currently under investigation for the treatment of CLI, and compare its output with Ficoll-based separation. The outputs of the MarrowStim P.A.D. Kit and Ficoll separation were characterized using an automated hematology analyzer, colony-forming unit (CFU) assays, and tubulogenesis assays. Hematology analysis indicated that the MarrowStim P.A.D. Kit concentrated the total nucleated cells, mononuclear cells, and granulocytes compared with baseline bone marrow aspirate. Cells collected were positive for VEGFR-2, CD3, CD14, CD34, CD45, CD56, CD105, CD117, CD133, and Stro-1 antigen. CFU assays demonstrated that the MarrowStim P.A.D. Kit output a significantly greater number of mesenchymal stem cells and hematopoietic stem cells compared with cells output by Ficoll separation. There was no significant difference in the number of endothelial progenitor cells output by the two separation techniques. Isolated cells from both techniques formed interconnected nodes and microtubules in a three-dimensional cell culture assay. This information, along with data currently being collected in large-scale clinical trials, will help instruct how different cellular fractions may affect the outcomes for CLI patients.

## Introduction

Critical limb ischemia (CLI) is a debilitating disease, which can lead to limb loss and is associated with high rates of morbidity and mortality. Treatment options for patients with CLI include surgical bypass and endovascular procedures. However, these treatment options have not led to durable results for all patients.^1^ Furthermore, 20–40% of patients with CLI are not eligible for traditional treatment due to the severity of their disease^2^ and amputation is often their only option due to severe pain and/or tissue necrosis.^3^ Patients with no-option CLI report have lower quality of life scores than patients with cancer, chronic heart disease, and chronic kidney disease.^4^ In patients who undergo a below-knee amputation, there is a 10% perioperative death rate and a 15% above-knee amputation rate within 1 year. After 2 years, there is a 30% death rate, 15% above-knee amputation rate, and a 15% contralateral limb amputation rate.^5^ These unsatisfactory outcomes have motivated the search for alternative approaches such as autologous cell therapies, which are currently under investigation as a potential treatment to address this unmet clinical need.

Bone marrow aspirate (BMA) has emerged as the most common cell source utilized in clinical studies evaluating the treatment of CLI with cell therapy. There has been debate regarding the cell types in BMA, which potentially drive tissue revascularization and angiogenesis.^6^ Bone marrow contains a complex mixture of cells, including hematopoietic stem cells, stromal stem cells, and their progeny. It has been hypothesized that stem cells play an important role in tissue regeneration either by directly contributing to new tissue formation or by acting as growth factor/cytokine delivery vehicles that orchestrate regeneration.^7,8^ Endothelial progenitor cells (EPCs) have also been explored as a cell source to treat CLI.^9^ However, stem and progenitor cells constitute a very small fraction of the total number of nucleated cells in BMA.^10^ Theoretically, these small numbers of stem and progenitor cells could divide, differentiate, and regenerate the target organ after transplantation.^11^ It may also be possible that more mature cell types drive the regeneration process. For example, mouse models of CLI demonstrated that purified blood monocyte populations were able to enhance angiogenesis.^12,13^ For the regeneration of complex tissues required in CLI, it is likely that a mixture of cells and signals are required to drive revascularization. Regardless of the cell types required, it is important that they be delivered in a volume appropriate for therapy.

Cell concentration has been utilized in most cell therapy approaches to treat CLI, to limit the volume injected into the muscle, and to prevent injury to the surrounding tissue. The volume of BMA harvested for CLI studies has ranged between 50 and 750 mL of bone marrow with a more recent emphasis on limiting the amount of bone marrow drawn to reduce the risks of anemia and heart failure.^14^ Several trials have used concentration devices and numerous trials have used Ficoll gradient centrifugation in a central laboratory.^14^ Although Ficoll gradient centrifugation has been a reliable laboratory technique to isolate the cellular fraction from bone marrow, it has several limitations for clinical use, including long processing and operating times, requirements for a hospital laboratory, requirements for multiple manual processing steps, which could affect the sterility of the product, and the need for multiple anesthesia or patient procedures to harvest and deliver the cells. Therefore, a system capable of delivering an equivalent isolation of the cellular fraction at the point of care would have the advantage of avoiding some of the limitations of the Ficoll gradient centrifugation method.

**FIG. 1.**
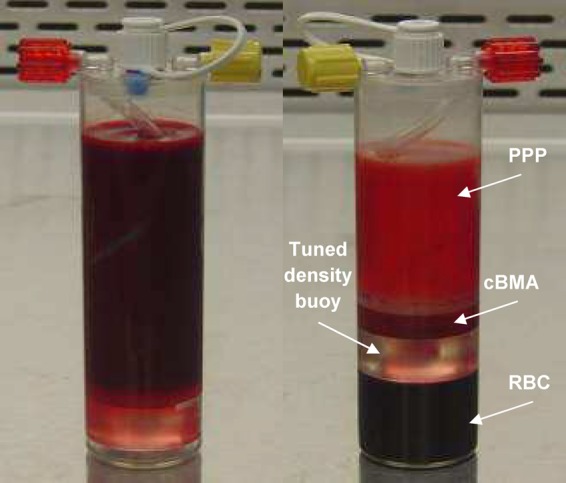
Sixty milliliters of bone marrow aspirate anticoagulated with Anticoagulant Citrate Dextrose Solution, Solution A was loaded into the concentration device (left). After centrifugation, the bone marrow is separated into platelet-poor plasma (PPP), concentrated bone marrow aspirate (cBMA), and red blood cells (RBCs) (right).

The goal of this study was to characterize the output of a device currently under investigation as a potential therapy for CLI (Fig. 1) and compare it with the output of Ficoll gradient centrifugation. Understanding the composition of the constituents in cell therapies will be important in furthering our understanding of the mechanisms that drive tissue regeneration and angiogenesis.

## Materials and Methods

### Bone marrow aspiration

BMA was collected from the iliac crest from healthy financially compensated subjects who qualified for blood donation as per AABB standards. Aspiration was performed by Poietics-Cambrex (Gaithersburg, MD), AllCells (Alameda, CA), and HemaCare (Van Nuys, CA) under their Institutional Review Board approval. The needle was repositioned during aspiration to maximize stem cell numbers and minimize dilution with peripheral blood. The samples were shipped overnight for testing.

### Bone marrow processing

One hundred milliliters of BMA was collected from each donor and combined with 20 mL of heparin (44 units/mL final concentration). Sixty milliliters of anticoagulated BMA was processed in the MarrowStim device (Biomet Biologics, Warsaw, IN) following the manufacturer's instructions for use. Approximately 6 mL of concentrated bone marrow aspirate (cBMA) was obtained; 0.5 mL of cBMA was used for complete blood count (CBC) analysis. Methods for Ficoll-Paque (GE Healthcare, Little Chalfont, United Kingdom) processing were followed according to the manufacturer's instructions. Briefly, 60 mL of anticoagulated BMA was diluted with 60 mL of phosphate-buffered saline (PBS; Invitrogen, Carlsbad, CA) +2% fetal bovine serum (FBS; ATCC, Manassas, VA). Eight milliliters of diluted BMA was layered on top of 4 mL of Ficoll-Paque in 15 separate conical tubes. Tubes were then centrifuged at 400 *g* for 30 min. After centrifugation, the buffy coat layer was collected and pooled into a conical tube and washed twice by diluting with PBS+2% FBS and centrifuging at 300 *g* for 10 min. Cells were finally diluted in 6 mL of PBS+2% FBS (same volume as MarrowStim device) before CBC analysis.

### Cell counts

CBCs were obtained using a hematology analyzer with a five-part differential (Cell-Dyn Sapphire; Abbott Laboratories, Dallas, TX; *n*=5 donors). The differential feature of the Cell-Dyn categorizes the cell population in the marrow into one of the five mature white blood cells (WBC) types (neutrophils, monocytes, lymphocytes, eosinophils, and basophils) based on cell size and granularity. Addition of the monocytes and lymphocytes together gives the mononuclear cell (MNC) fraction in the marrow.

A protocol shown to provide accurate cell counts in platelet-rich plasma samples from whole blood was used to acquire cell counts.^15^ In brief, counts were taken in triplicate from samples placed on a rocker (Ames Aliquot Mixer Model 4651; Ames Company, Elkhart, IN) for a minimum of 15 min before counting to allow for an even distribution of the cells within the sample.

### Flow cytometry

BMA and cBMA samples (*n*=6 donors) were labeled as per manufacturer's instructions for CD105 (Abcam, Cambridge, MA), VEGFR-2 (R&D Systems, Minneapolis, MN), and CD3, CD14, CD34, CD45, CD56, CD117, CD133, and Stro-1 (BD Biosciences, San Jose, CA). Labeled cells were washed with fluorescence-activated cell sorting (FACS) buffer (PBS solution containing 0.5% bovine serum albumin and 0.1% sodium azide), pelleted, and fixed in 0.5% paraformaldehyde and 0.1% sodium azide in PBS solution. Samples were then analyzed using an FACSCalibur device (BD Biosciences). Ten thousand events were acquired and analyzed using commercially available software. The concentration of labeled cells in each sample was calculated by multiplying the percent detected by the concentration of WBCs in that sample as measured by the automated cell counting method described above.

### Colony-forming unit assays

For the colony-forming unit–fibroblast (CFU-F) assay (mesenchymal stem cells [MSCs]), 2.5 × 10^5^ MNCs were plated in duplicate in six-well plates in Mesencult media (Stem Cell Technologies, Vancouver, Canada). Cultures were incubated at 37°C, 5% CO_2_, for 14 days before being fixed in 100% methanol and stained with Giemsa staining solution (Sigma-Aldrich, St. Louis, MO). Colonies were counted microscopically at 50× magnification. Only colonies with >40 cells were counted.

For the CFU-Hill assay (EPC), 5 × 10^6^ MNCs were plated onto six-well fibronectin-coated plates (Corning, Corning, NY) and incubated in a 37°C, 5% CO_2_ incubator. On day 2, nonadherent cells were collected and plated onto 24-well fibronectin-coated plates at 10^6^ cells per well. Cultures were incubated for an additional 3 days before being fixed with methanol (Sigma, St. Louis, MO) and stained with Giemsa staining solution. CFU-Hill colonies were defined as a central core of round cells with radiating, elongated spindle-like cells at the periphery and were counted microscopically at 50× magnification.

For the CFU-granulocyte, erythrocyte, macrophage, megakaryocyte (CFU-GEMM) assay (hematopoietic stem cells), cells were prepared at 5 × 10^5^ nucleated cells in PBS with 2% FBS. Then, 0.3 mL of this cell solution was added into 3 mL of MethoCult^®^ methylcellulose-based media (Stem Cell Technologies). After bubbles dissipated, 1.1 mL of the cell–media solution was pipetted into 35-mm Petri dishes (*n*=2; Stem Cell Technologies). A third Petri dish was filled with sterile water and left uncovered. All three Petri dishes were placed in a 100-mm Petri dish (Corning) and placed in an incubator at 37°C, 5% CO_2_, in air for 14 days. Following the incubation, each dish was placed on a grid and colonies were counted at 50× magnification. CFU-GEMM colonies were defined as a dense core of erythroid clusters and recognizable granulocyte and macrophage cells at the periphery.

### Microtubule and node assay

MNCs were plated onto T-75 flasks (Fisher Scientific, Fairlawn, NJ) in Mesencult media and washed once the next day before incubating in Mesencult media at 37°C, 5% CO_2_. Cells were then harvested and seeded onto a 24-well plate coated with Matrigel (BD Biosciences). Cells were cultured for 16 h and stained with calcein AM (Corning) before photographing representative areas and counting the nodes at 50× magnification. Node counts were averaged between all images.

### Statistical analysis

Data are presented as mean±standard deviation. Statistical significance between population means was compared using Student's *t*-test (α=0.05).

## Results

### Hematology analyzer cell counts

**FIG. 2.**
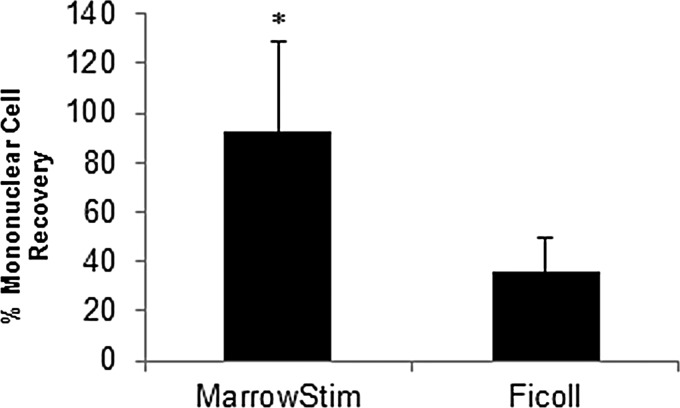
Percent mononuclear cell recovery in the output of the MarrowStim P.A.D. Kit and Ficoll separation. *n* = 5, *p < 0.05.

CBC analysis was performed on baseline BMA, Ficoll-purified marrow, and cBMA. Cellular components of the marrow were categorized based on size and granularity into one of the five mature WBC types on the hematology analyzer (Table 1). Cells were concentrated significantly with the MarrowStim P.A.D. Kit compared with the Ficoll method (Table 1). The MarrowStim P.A.D. Kit provided an average of 233,000 cells/μL or 1.4 × 10^9^ total nucleated cells. Of these cells, 26% were MNCs. The percent recovery of MNCs was significantly greater than the output of the MarrowStim device compared with Ficoll separation (*p*=0.007; Fig. 2). The standard Ficoll method did remove more red blood cells compared with the concentration device (Table 1).

**Table 1.** **Comparison of CBC Analysis on BMA, Ficoll-Separated Marrow, and cBMA from the MarrowStim P.A.D. Kit (*n* = 5)**

	**TNC (k/μL)**	**MONO (k/μL)**	**LYM (k/μL)**	**NEU (k/μL)**	**EOS (k/μL)**	**BASO (k/μL)**	**PLT (k/μL)**	**RBC (M/μL)**
Preparation								
BMA	23 ± 7	1 ± 0	5 ± 1	17 ± 6	0 ± 0	0.1 ± 0.0	117 ± 26	4 ± 1
Ficoll	53 ± 20	3 ± 2	9 ± 8	27 ± 16	4 ± 7	0.7 ± 0.4	90 ± 34	0 ± 0
MarrowStim	233 ± 61	10 ± 2	51 ± 11	160 ± 49	11 ± 16	1.2 ± 0.6	753 ± 233	3 ± 2
Fold increase from BMA								
Ficoll	2.3	2.4	3.9	1.6	N/A	10.6	0.8	0.03
MarrowStim	10	8.1	10.6	9.4	N/A	19.4	6.4	0.7

BASO, basophil; BMA, bone marrow aspirate; CBC, complete blood count; cBMA, concentrated bone marrow aspirate; EOS, eosinophil; LYM, lymphocyte; MONO, monocyte; N/A, not available due to zero value in input BMA; NEU, neutrophil; PLT, platelet; RBC, red blood cell; TNC, total nucleated cells.

### Flow cytometry

There were no significant changes in CD marker expression pre- or postprocessing (Table 2), except for an increase in the VEGF receptor (*p*=0.04). Of the CD markers analyzed, the marker for WBC (CD45) was most prevalent. Due to the significant increase in cellular concentration, there was a significant increase in the concentration of cells that were antigen positive for the markers tested.

**Table 2.** **FACS Analysis on BMA and cBMA from the MarrowStim P.A.D. Kit (*n* = 6)**

**CD marker**	**Function and cell type**	**BMA (%)**	**cBMA (%)**
R1 gate	Lymphocytes	17.4 ± 6.6	20.5 ± 3.5
R2 gate	Monocytes	4.0 ± 1.2	4.3 ± 1.5
R3 gate	Granulocytes	75.5 ± 5.8	71.2 ± 7.2
VEGFR-2	Tyrosine kinase receptor (endothelial cells and precursors)	5.6 ± 1.8	7.5 ± 3.2
CD3	Antigen receptor (T cells)	9.0 ± 3.3	11.5 ± 2.9
CD14	Surface glycoprotein (monocytes and macrophages)	5.4 ± 2.5	4.8 ± 3.3
CD34	Transmembrane glycoprotein (hematopoietic and endothelial progenitor cells)	0.9 ± 1.0	1.0 ± 0.6
CD45	Transmembrane protein tyrosine kinase (white blood cells and hematopoietic stem cells)	91.1 ± 4.9	90.7 ± 6.6
CD56	NCAM-1 (heparin-binding glycoprotein; T cells)	8.3 ± 8.5	7.7 ± 5.8
CD105	TGF-β receptor, endoglin (SH2; endothelial cells, monocytes, macrophages, a subpopulation of hematopoietic stem cells, and cultured mesenchymal stem cells)	4.1 ± 3.4	9.3 ± 16.1
CD117	Tyrosine kinase receptor (hematopoietic stem cells, B cells, and T cells)	3.2 ± 5.5	1.8 ± 1.2
CD133	Trasmembrane protein (hematopoietic stem cells and endothelial cells)	0.2 ± 0.1	0.2 ± 0.1
Stro-1	Bone marrow stromal cells	0.8 ± 0.6	1.1 ± 0.6

There were no significant differences between the BMA and the cBMA for all markers analyzed, except for the VEGFR-2 receptor (*p* = 0.04).

FACS, fluorescence-activated cell sorting; TGF-β, transforming growth factor-β.

### CFU assays

**FIG. 3.**
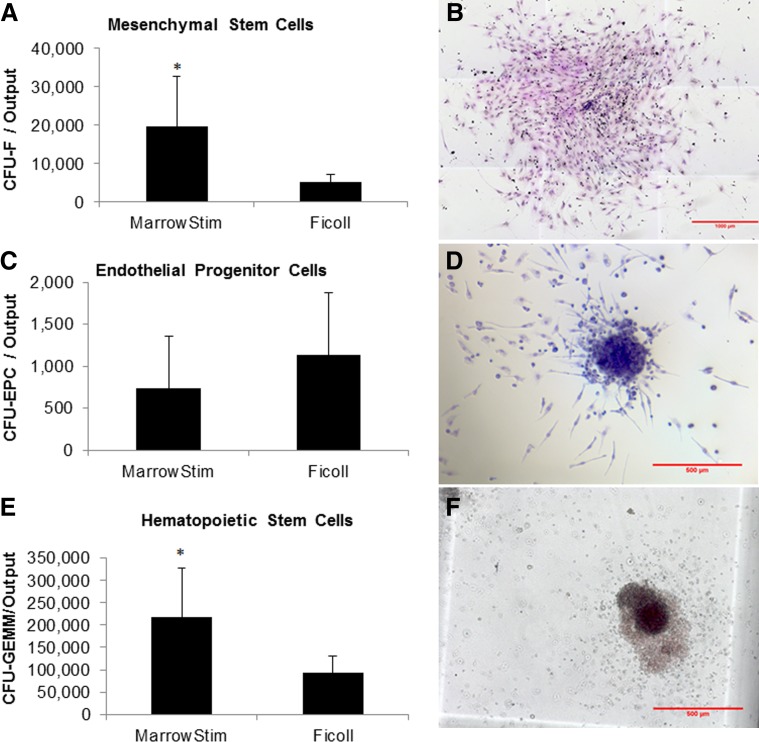
Colony-forming units in the output of the MarrowStim P.A.D. Kit and Ficoll processing, as well as representative photomicrographs. Colony-forming unit–fibroblast (CFU-F) **(A, B)**, colony-forming unit–endothelial progenitor cells (CFU-EPCs) **(C, D)**, and colony-forming unit–granulocyte, erythrocyte, macrophage, megakaryocyte (CFU-GEMM) **(E, F)**. *n* = 5, *p < 0.05.

The clonal capacity of the marrow aspirate cells after processing with the MarrowStim P.A.D. Kit and Ficoll separation was analyzed (Fig. 3). There were significantly greater numbers of CFU-Fs (MSCs; *p*=0.033) and CFU-GEMMs (myeloid progenitor cells; *p*=0.026) in the output of the MarrowStim P.A.D. Kit compared with Ficoll separation (Fig. 3A, 3E). The CFU-F concentration in the output of the MarrowStim P.A.D. Kit was 3274±2159 CFU-Fs/mL. There was no significant difference in the number of CFU-endothelial progenitor cells (CFU-EPCs) in the output of the two systems (*p*=0.12; Fig. 3C). Representative photomicrographs of CFU-F, CFU-EPC, and CFU-GEMM colonies are shown in Figure 3B, D, and F, respectively.

### Microtubule and node assay

**FIG. 4.**
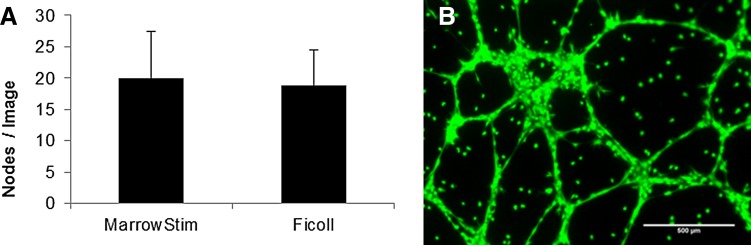
Characterization of cells' ability to form nodes and microtubules after isolation from the MarrowStim P.A.D. Kit and Ficoll separation. **(A)** Equal numbers of cells output from the MarrowStim P.A.D Kit and Ficoll separation formed an average number of nodes that were not statistically different (*p* > 0.05). **(B)** Representative fluorescent micrograph demonstrating that cells isolated by the MarrowStim separator form nodes and microtubules in 16 h.

Cells from the MarrowStim P.A.D. Kit and Ficoll separation rapidly formed microtubules and nodes within 16 h (Fig. 4). The number of nodes per image was not statistically different when the same number of MNCs from the MarrowStim P.A.D. Kit and Ficoll separation were cultured on Matrigel (Fig. 4A). A representative fluorescent micrograph of the nodes from MNCs from the MarrowStim P.A.D. Kit is shown in Figure 4B.

## Discussion

The results of this study demonstrated that the MarrowStim P.A.D. Kit could replace the time-consuming and labor-intensive Ficoll separation technique and provide for point-of-care processing of autologous cBMA. Specifically, the total nucleated cells (TNCs) (*p*=4E^−7^), MNCs (*p*=3E^−7^), and granulocytes (*p*=2E^−6^) were significantly increased by the MarrowStim P.A.D. Kit compared with Ficoll separation (Table 1 and Fig. 2). This concentration effect was also confirmed through analysis of cell surface markers for key cell types in BMA and cBMA (Table 2). Increases in the number of CFUs for MSCs, EPCs, and hematopoietic progenitor cells demonstrated that the MarrowStim P.A.D. Kit could concentrate rare stem and progenitor cells in bone marrow (Fig. 3). Finally, cells isolated by the MarrowStim P.A.D. Kit formed microtubules and formed interconnected nodes and microtubules in a three-dimensional cell culture environment (Fig. 4).

These experiments demonstrated that the MarrowStim P.A.D. Kit could concentrate both mature and progenitor bone marrow cell types, which may play a role in the revascularization process. Limitations in this study include a relatively small sample number (*n*=5–7 donors depending on the experiment) and no autologous therapies have yet been proven safe and effective in the treatment of CLI. However, the results of this study serve as a reference point as to the cellular profile in the output of the device currently under investigation, which can be later used to evaluate success or failure of trials and put into context of other cell therapies for CLI.

The mechanism by which cells affect the revascularization process in CLI is still under investigation and no cell therapy has yet proven safe and effective for the treatment of CLI. Teams have begun to explore which cell types in cBMA could affect the revascularization process individually. Culture-expanded endothelial cells have been explored as a potential cell source for CLI due to their ability to differentiate into multiple somatic cell types and their capacity to secrete proangiogenic factors.^9^ Culture-expanded MSCs from bone marrow,^16^ adipose tissue,^17^ and induced pluripotent stem cells^18^ have all been explored in rodent studies to treat CLI. Ficoll separation and other separators have been used to treat rodent models of CLI. Due to the nature of the MarrowStim P.A.D. Kit, which required 60 mL of autologous BMA per device, rodent studies were not possible without using athymic animals. Removing the immune response component of revascularization might result in a very different response to cBMA from the MarrowStim P.A.D. Kit and Ficoll separation. In the future, large animal and human studies may instruct how autologous cBMA affects the CLI disease process.

Several pre-clinical studies have suggested that monocytes play a critical role in directing revascularization.^12,13^ Monocytes contributed to a small, but measurable, fraction in the output in this study (10±2%). Studies are ongoing to correlate cell counts with clinical results in CLI trials.^19^ Such information could enable a better understanding of the mechanism of action of cBMA in treating CLI and provide general information for future regenerative medicine approaches that require vascularization.

One potential advantage of utilizing a BMA as a cell source is that a variety of cell types are available to be concentrated. In addition to MSCs, the cBMA in this study contained EPCs and hematopoietic stem cells. EPCs and hematopoietic stem cells can present human leukocyte antigens,^20,21^ so these cells must be depleted in an allogeneic live or cadaveric stem cell source. While it is unclear what exact cell population is required to treat CLI, EPCs and hematopoietic stem cells are critical for angiogenesis and vasculogenesis.^22–25^ In addition, MSCs and hematopoietic stem cells have been shown to cross talk and create a stem cell niche that is beneficial for both stem cell types.^26,27^ Soluble factors released from one progenitor cell type to another have been shown to be required for cell survival and proliferation of colonies.^28^ This cross talk between cell types could play a role in the efficacy of future cell therapies and suggests that a stem cell milieu might be advantageous over an isolated or purified cell population.

Concentrating bone marrow with a disposable device such as the MarrowStim P.A.D. Kit has several pragmatic advantages over Ficoll separation and, if first proven safe and effective, could enable earlier or concurrent intervention when treating CLI. In a pilot study testing cBMA for the treatment of CLI, cBMA was formed using Ficoll separation techniques for the first 14 patients, which required two procedures (one for bone marrow harvest and one for cell delivery). The MarrowStim P.A.D. Kit was used for the next 15 patients to decrease procedure time and allow patients to undergo a single procedure. Switching from Ficoll separation to the MarrowStim P.A.D. Kit decreased the average procedure time from 527±39 min to 114±19 min.^29^ Significantly, treatment with the MarrowStim P.A.D. Kit does not prohibit other options that might become necessary or available in the future. A pivotal clinical trial is currently ongoing to evaluate the effectiveness of the MarrowStim P.A.D. Kit to prevent or delay amputation in patients with CLI (http://clinicaltrials.gov/ct2/show/NCT01049919). Additional studies are also being designed to further explore the potential role of the MarrowStim P.A.D. Kit in the peripheral arterial disease/CLI patient population.

In conclusion, the MarrowStim P.A.D. Kit efficiently concentrated cell types from BMA that may have a role in inducing new vascularization in patients with CLI. This disposable device was more efficient at capturing TNCs, MNCs, and granulocytes than using traditional Ficoll separation techniques. CFU assays indicated that rare stem and progenitor cells were captured and concentrated by the MarrowStim P.A.D. Kit. This preliminary research will require confirmation in a larger set of samples and correlation with clinical outcomes to determine which cell type or types drive the potential of the MarrowStim P.A.D. Kit to treat CLI. As cellular therapies continue to emerge, it will be important to quantify the source, type, and concentrations of cells used for a given therapy. Standardization between the outcomes of characterization methods is needed. Without a consensus on stem cell characterization methods, it will be challenging to draw conclusions between various clinical studies. Research is ongoing to advance the results of this study and correlate the cellular profile of the MarrowStim P.A.D. Kit and clinical success when treating CLI.

## Abbreviations Used

BMA: bone marrow aspirate
CBC: complete blood count
cBMA: concentrated bone marrow aspirate
CFU: colony-forming unit
CFU-EPC: CFU-endothelial progenitor cells
CFU-F: CFU-fibroblast
CFU-GEMM: CFU-granulocyte, erythrocyte, macrophage, megakaryocyte
CLI: critical limb ischemia
EPC: endothelial progenitor cell
FACS: fluorescence-activated cell sorting
FBS: fetal bovine serum
MNC: mononuclear cell
MSC: mesenchymal stem cell
PBS: phosphate-buffered saline
RCF: relative centrifugal force
TGF-β: transforming growth factor-β
WBC: white blood cell
